# Development of a Colloidal Gold Kit for the Diagnosis of Severe Fever with Thrombocytopenia Syndrome Virus Infection

**DOI:** 10.1155/2014/530621

**Published:** 2014-04-14

**Authors:** Xianguo Wang, Quanfu Zhang, Fen Hao, Xunian Gao, Wei Wu, Minyao Liang, Zhihua Liao, Shuhong Luo, Weiwen Xu, Dexin Li, Shiwen Wang

**Affiliations:** ^1^Institute of Antibody Engineering, School of Biotechnology, Southern Medical University, 1838 N. Guangzhou Avenue, Guangzhou 510515, China; ^2^Key Laboratory for Medical Virology, MOH, National Institute for Viral Disease Control and Prevention, China CDC, 155 Changbai Road, Beijing 102206, China; ^3^DaAn Gene Co. Ltd. of Sun Yat-sen University, 19 Xiangshan Road, Guangzhou 510515, China

## Abstract

It is critical to develop a cost-effective detection kit for rapid diagnosis and on-site detection of severe fever with thrombocytopenia syndrome virus (SFTSV) infection. Here, an immunochromatographic assay (ICA) to detect SFTSV infection is described. The ICA uses gold nanoparticles coated with recombinant SFTSV for the simultaneous detection of IgG and IgM antibodies to SFTSV. The ICA was developed and evaluated by using positive sera samples of SFTSV infection (*n* = 245) collected from the CDC of China. The reference laboratory diagnosis of SFTSV infection was based on the “gold standard”. The results demonstrated that the positive coincidence rate and negative coincidence rate were determined to be 98.4% and 100% for IgM and 96.7% and 98.6% for IgG, respectively. The kit showed good selectivity for detection of SFTSV-specific IgG and IgM with no interference from positive sera samples of Japanese encephalitis virus infection, Dengue virus infection, Hantavirus infection, HIV infection, HBV surface antigen, HCV antibody, *Mycobacterium tuberculosis* antibody, or RF. Based on these results, the ICS test developed may be a suitable tool for rapid on-site testing for SFTSV infections.

## 1. Introduction

Severe fever with thrombocytopenia syndrome (SFTS) is an emerging infectious disease recently identified in central and northeast China. It is caused by a novel SFTS bunyavirus (SFTSV), in the genus of* Phlebovirus*, family Bunyaviridae [1–3]. Recently, 15 provinces in China have reported cases of SFTS. The case fatality rate of this disease initially was 30% and remained to an average of 10% currently [1, 4, 5]. The clinical manifestations of SFTS include high fever, gastrointestinal symptoms, thrombocytopenia, leukocytopenia, multiorgan dysfunction, and hemorrhagic tendency in severe cases [2]. Clinical presentations of SFTS, however, are less specific and need to be differentiated from various infectious diseases, particularly from hemorrhagic fever with renal syndrome (HFRS) caused by Hantavirus and human anaplasmosis [6].

At present, SFTSV detection methods involve the detection of IgM and IgG by enzyme-linked immunosorbent assays (ELISA), an SFTSV neutralization test, virus isolation, and quantitative reverse-transcriptase polymerase chain reaction (qRT-PCR) with high specificity and sensitivity [1, 7, 8]. This provides an effective means for diagnosis and timely treatment of the SFTS. However, the disease often occurs in mountainous regions with poor medical conditions where the application of these methods is limited due to the required technical expertise and the necessity of specialized laboratory equipment or facilities. As SFTS is an infectious disease, early diagnosis is critical for effective control of the disease. As such, it is necessary to develop a rapid, on-site testing and easily performed assay for the detection of SFTSV infections.

The ICA is a new technique in which a colloidal gold-labeled antigen or antibody is used as a tracer to detect antibody or antigen, respectively. This type of assay has been applied widely. A kit based on ICA should be a reliable and rapid method for clinical diagnosis and, even in the hands of inexperienced personnel, easy to perform under harsh field conditions. The aim of this study was to conduct an evaluation of the performance of the rapid ICA based on the antibody capture format for SFTSV-specific IgG and IgM using laboratory confirmed sera from patients with SFTSV and control sera from SFTSV-free individuals.

## 2. Materials and Methods

### 2.1. Antigen

Recombinant nucleocapsid protein (NP) of SFTSV was prepared as described previously [9]. Briefly, the S gene of the SFTSV strain HB29 was cloned into pET30A vector, expressed in* E. coli,* purified through His-Tag affinity chromatography purification, and kept in −20°C for further use.

### 2.2. The Main Raw Materials

Anti-human IgM antibody and anti-human IgG antibody were purchased from BiosPacific. Streptavidin was purchased from Sigma. Biotin-BSA was purchased from YouDi BioTechnology Co. Ltd. of Guangzhou.

### 2.3. “Gold Standard”

The reference laboratory diagnosis of SFTSV infection was based on the “gold standard.” Referring to “Fever with Thrombocytopenia Syndrome Prevention Guide (2010 version)” issued from the Office of the Ministry of Health of China. The “gold standard” of laboratory-confirmed SFTSV infection by the use of real-time PCR and/or Indirect fluorescent antibody test (IFAT) [9, 10]. SFTSV nucleic acid was detected in specimens from patients using real-time quantitative PCR nucleic acid detection kit approved by China Food and Drug Administration (CFDA) (CFDA Registration number 340166, China) and/or fourfold increase of virus-specific IgG in paired sera using Indirect fluorescent antibody test (IFAT) as the ultimate basis for determining the sample infected with SFTSV and the presence of SFTSV-specific IgM in the sample; IFAT test results to determine whether the presence of SFTSV-specific IgG in the sample.

### 2.4. Preparation of Colloidal Gold

Colloidal gold particles with a mean diameter of 30 nm were prepared using a modified Frens method [11, 12]. Under rapid magnetic stirring, 2.36 mL of 1% trisodium citrate solution (w/v) was added to 100 mL of the aqueous solution containing 0.0164 g chloroauric acid (HAuCl_4_) at 100°C and boiled for 5 min. After boiling for 10 minutes, the colloidal gold was gradually cooled.

### 2.5. Colloidal Gold-Recombinant SFTSV and Colloidal Gold-Streptavidin Conjugates

The pH of the solution was adjusted by addition of 0.1 M K_2_CO_3_. For recombinant SFTSV, 200 *μ*L 0.1 M K_2_CO_3_ was added to 10 mL colloidal gold. Recombinant SFTSV (232 *μ*g) was added dropwise to 10 mL of pH-adjusted colloidal gold solution. For streptavidin, 50 *μ*L 0.1 M K_2_CO_3_ was added to 10 mL colloidal gold. Streptavidin (230 *μ*g) was added dropwise to 10 mL of pH-adjusted colloid gold solution. The mixture was stirred vigorously for 40 minutes; 100*μ*L of 20% (w/v) BSA was added to block excess reactivity of the gold colloid. The mixture was stirred for additional 20 minutes. After the mixture was centrifuged twice at 12,000 rpm (4°C for 20 minutes) to remove the uncoordinated protein, the final pellets were resuspended in 50 *μ*L of 0.02 M Tris-HCl buffer (pH 8.0) containing BSA (1%, w/v), NaN_3_ (0.02%, w/v), sucrose (20%, w/v), and trehalose (5%, w/v).

### 2.6. Preparation of the Immunochromatographic Strip (ICS)

The immunochromatographic strip includes four components: a sample pad, a conjugate pad, a nitrocellulose membrane, and an absorbent pad. Anti-human IgM antibody, anti-human IgG antibody, and Biotin-BSA dissolved in PBS were loaded onto the test and control lines on the nitrocellulose membrane using an XYZ3060 Dispense Workstation (BioDot, Inc., Sky Park, Irvine, CA) and dried for two hours at 37°C. The colloidal gold-recombinant SFTSV and gold-streptavidin were sprayed onto a glass-fiber membrane with an XYZ3060 Dispense Workstation. The sample pad, the pretreated conjugate pad, the nitrocellulose membrane, and the absorbent pad were adhered to a backing plate (300 mm × 60 mm) in the proper order. The plate was then cut into 3 mm wide strips using a strip cutter. The strips were stored under dry conditions until use.

### 2.7. The Best Conditions of the ICS

First, the colloidal gold-streptavidin was sprayed onto a glass-fiber membrane with OD_540_ = 10 at a rate of 0.8 *μ*L/mm and Biotin-BSA was loaded onto the control line on the nitrocellulose membrane with 2 mg/mL at a rate of 0.1 *μ*L/mm. It is chosen by good color effect in our previous experimental results. Second, the colloidal gold-recombinant SFTSV was sprayed onto a glass-fiber membrane with OD_540_ = 45 at a rate of 0.4 *μ*L/mm, 0.6 *μ*L/mm, 0.8 *μ*L/mm, and 1.0 *μ*L/mm. The anti-human IgM antibody was loaded onto the M line on the nitrocellulose membrane with 1 mg/mL, 2 mg/mL, and 3 mg/mL at a rate of 0.1 *μ*L/mm. The anti-human IgG antibody was loaded onto the G line on the nitrocellulose membrane with 1 mg/mL, 2 mg/mL, and 3 mg/mL at a rate of 0.1 *μ*L/mm.

### 2.8. Sensitivity of the ICS Test

The sensitivity of IgG was evaluated by testing a series of 2-fold dilutions of an IgG positive sera sample with antibody titer of 640 tested by IFAT in PBS. The sensitivity of IgM was evaluated by testing a series of 2-fold dilutions of a positive sera sample with 1.77 × 10^6^ TCID50/mL in PBS. Each dilution was then added to the ICS test strip, and the sensitivity was determined by finding the end point dilution.

### 2.9. Specificity of the ICS Test

To evaluate the cross-reactivity of the ICS test strip, positive sera samples of SFTSV infection, Japanese encephalitis virus infection, Dengue virus infection, Hantavirus infection, HIV infection, HBV surface antigen, HCV antibody,* Mycobacterium tuberculosis* antibody, and RF were tested with the test strip. SFTSV was used as the positive control and PBS was used as the negative controls.

### 2.10. Empirical Detection of Clinical Samples

A total of 1082 clinical samples were collected and laboratory confirmed by China CDC (245 from patients with SFTSV, 58 from HFRS, 112 from influenza virus infection, 10 from HIV infection, 10 from HBV infection, 10 from HCV infection, 10 from RF, 38 from common flu virus infection, and 589 general population samples). All samples were detected and validated with both “gold standard” methods and our assay by CDC of China. These studies were reviewed and approved by the regional ethics committee.

## 3. Results

### 3.1. The Principle and Procedure of the Immunochromatographic Assay

**Figure 1:**
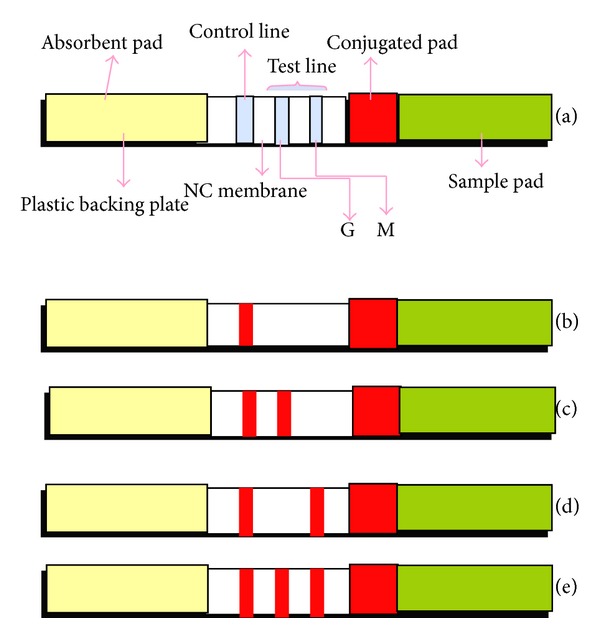
(a) A schematic representation of the immunochromatographic assay; the NSI-strip included five components: three pads (sample, conjugate, and absorbent), an NC membrane, and a plastic backing plate. The conjugate pad contained dried nanogold-Ab probe, which provided an easily visible color owing to the red color itself. There were three lines on the NC membrane: the control line and test lines (G and M). The control line contained Biotin-BSA; the G test zone included anti-human IgG antibody and the M test zone included anti-human IgM. (b) The result of negative detection, (c) the result of IgG positive detection, (d) the result of IgM positive detection, and (e) the result of both IgG and IgM positive detection.

In the detection test, the sample diffused rapidly through the conjugate pad when the liquid sample was added to the sample hole of the assay device. For positive samples, one or two lines in the test region appeared if the sample contained IgM or/and IgG, respectively. Accordingly, the presence of a colloidal gold-labeled recombinant SFTSV caused the appearance of a red line in the test region. For negative samples a colloidal recombinant SFTSV did not form in the test region due to the absence of IgM and IgG and therefore no red line appeared. In the control region, a red line appeared due to the formation of a colloidal gold-labeled streptavidin. Thus, the appearance of two or three red bands within 10 minutes (one line in the control region and one or two lines in the test region) indicates a positive result, whereas the appearance of only one red line in the control region indicates a negative result (Figure 1). All samples were diluted by 1 : 100 using PBS before experiment.

### 3.2. The Best Conditions of the ICS

To determine the best conditions of the colloidal gold-recombinant SFTSV and the anti-human IgM antibody, three negative samples, three positive samples, and three kinds of specific samples (RF, HBV, and HCV) were tested under different dilutions. The results are presented in Table 1. The results indicated that the best conditions of the colloidal gold-recombinant SFTSV were 0.8 *μ*L/mm and the anti-human IgM antibody was 2 mg/mL. To determine the best conditions of the anti-human IgG antibody, three negative samples and three positive samples were tested under different dilutions. The results are presented in Table 2 indicating that the best condition of the anti-human IgG antibody was 2 mg/mL.

### 3.3. Sensitivity of the ICS Test

To determine the sensitivity of IgG and IgM, a series of 2-fold dilutions of IgG positive sera sample and IgM positive sera samples were tested. The results of the sensitivity of IgG were 1 : 512 and the sensitivity of IgM was 1 : 128 (Table 3).

### 3.4. Specificity of the ICS Test

**Figure 2:**
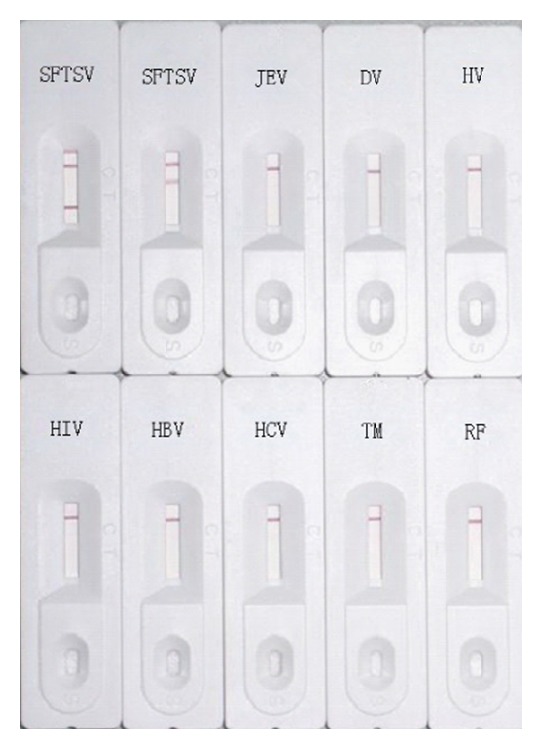
The specificity detection of the immunochromatographic strips for positive sera samples of IgM of SFTSV infection, IgG of SFTSV infection, Japanese encephalitis virus infection, Dengue virus infection, Hantavirus infection, HIV infection, HBV surface antigen, HCV antibody,* Mycobacterium tuberculosis* antibody, and RF, respectively.

To determine the specificity of the ICS, positive sera samples of SFTSV infection, Japanese encephalitis virus infection, Dengue virus infection, Hantavirus infection, HIV infection, HBV surface antigen, HCV antibody,* Mycobacterium tuberculosis* antibody, and RF were simultaneously tested. All specimens other than SFTSV infection resulted in one strong band on the control line. Samples positive for SFTSV infection resulted in the appearance of an additional band on the test line M or/and G of the ICS test (Figure 2). Thus, the results indicated that the immunochromatographic strip test has high specificity for SFTSV.

### 3.5. Experimental Detection of Clinical Samples

The 1082 clinical samples were analyzed using the “gold standard” and the ICS. Of the 1082 clinical samples, 241 IgM antibody positives and 841 negatives were obtained with the ICS, whereas 245 positives and 837 negatives were detected by “gold standard;” 179 IgG antibody positives and 903 negatives were obtained with the ICS, whereas 171 IgG antibody positives and 911 negatives were detected by “gold standard.” Thus, compared with the “gold standard,” the positive coincidence rate and negative coincidence rate were determined to be 98.4% and 100% for IgM (Table 4) and 96.7% and 98.6% for IgG (Table 5), respectively. There was excellent agreement between the ICS results and the “gold standard” with Kappa_IgM_ = 0.989 and Kappa_IgG_ = 0.945.

## 4. Discussion

SFTSV is an emerging pathogen which could cause serious disease in humans. The case fatality ratio in laboratory-confirmed patients was very high. Causes of death were cerebral hemorrhage or multiorgan failure [1, 3, 13]. It is important to develop an accurate and efficient method for SFTSV detection. Based on data from an epidemiological investigation, of all SFTSV infection cases, over 97% were farmers working and living in remote villages. The medical resources in these regions are limited. A rapid, accurate, and low-cost method for SFTSV detection is very necessary.

ICA is very promising to be used for the identification of analytes and pathogens at the point of care and home use, because it provides relatively fast assay, minimally trained personnel, low cost, and ability to operate in regions where sophisticated laboratory facilities are not available, and now ICA has been widely used for rapid detection of toxic or harmful substances in many fields such as food safety monitoring [14–17] and point-of-care diagnostics [18–21]. The ICA we developed is a new detection method with gold nanoparticles coated with recombinant SFTSV for the simultaneous detection of IgG and IgM antibodies to SFTSV. It is a convenient, rapid, accurate, and low-cost method for SFTSV detection.

The results of the ICA in this study were consistent with results obtained from gold standard. The ICA was applied in detection of SFTSV in 1082 clinical samples collected from the CDC of China. Compared with the “gold standard,” the positive coincidence rate and negative coincidence rate were determined to be 98.4% and 100% for IgM and 96.7% and 98.6% for IgG, respectively. There was excellent agreement between the ICS results and the “gold standard” with Kappa_IgM_ = 0.989 and Kappa_IgG_ = 0.945.

The results of the specificity test showed that the ICA was specific for the detection of IgG and IgM of SFTSV. There is no cross-reaction with Japanese encephalitis virus infection, Dengue virus infection, Hantavirus infection, HIV infection, HBV surface antigen, HCV antibody,* Mycobacterium tuberculosis* antibody, or RF.

In conclusion, the ICA for detection of IgG and IgM of SFTSV is cheap and easy to use with rapid and accurate results. In particular, the ICA demonstrated high specificity and sensitivity without the requirement for very specific and expensive equipment and complicated techniques. All the advantages of this ICA make it suitable and useful for detection of SFTSV in the field epidemic survey.

## 5. Conclusion

In this study, an ICA using gold nanoparticles coated with recombinant SFTSV for the simultaneous detection of IgG and IgM antibodies to SFTSV was successfully developed. It was the first time that test strip based assay was applied for the detection of SFTSV infections. The positive coincidence rate and negative coincidence rate of the ICS were determined to be 98.4% and 100% for IgM and 96.7% and 98.6% for IgG, which suggested that this method was sensitive enough for detecting SFTSV infections. Moreover, it also showed good selectivity for detection of IgG and IgM antibodies of SFTSV with no interference from Japanese encephalitis virus infection, Dengue virus infection, Hantavirus infection, HIV infection, HBV surface antigen, HCV antibody,* Mycobacterium tuberculosis* antibody, and RF. Furthermore, it is a rapid, on-site testing and easily performed assay. It is very beneficial for its applications in the mountainous and hilly areas with poor medical condition and it can control the spread of disease in endemic areas timely. In summary, this study demonstrated that this method is rapid, sensitive, cost-effective, and convenient for the on-site detection of SFTSV infections.

## Figures and Tables

**Table 1:** The results of different conditions of the colloidal gold-recombinant SFTSV and the anti-human IgM antibody.

Anti-human IgM antibody	Colloidal gold-recombinant SFTSV (*μ*L/mm)	Negative samples			Positive samples			Specific samples		
		A	B	C	1	2	3	RF	HBV	HCV
1 mg/mL	0.4	−	−	−	−	−	−	−	−	−
	0.6	−	−	−	−	−	−	−	−	−
	0.8	−	−	−	−	−	−	−	−	−
	1.0	−	−	−	−	−	+	−	−	−

2 mg/mL	0.4	−	−	−	−	−	+	−	−	−
	0.6	−	−	−	+	−	+	−	−	−
	0.8	−	−	−	+	+	+	−	−	−
	1.0	−	−	−	+	+	+	+	−	−

3 mg/mL	0.4	−	−	−	−	+	+	+	−	−
	0.6	−	−	−	−	+	+	+	−	−
	0.8	−	−	+	+	+	+	+	−	−
	1.0	−	−	+	+	+	+	+	+	−

**Table 2:** The results of different conditions of the anti-human IgG antibody.

Anti-human IgG antibody	Negative samples			Positive samples		
	RF	HBV	General population sera samples	4	5	6
1 mg/mL	−	−	−	−	−	−
2 mg/mL	−	−	−	+	+	+
3 mg/mL	+	+	−	+	+	+

**Table 3:** The results of sensitivity of IgG and IgM.

	1 : 2	1 : 4	1 : 8	1 : 16	1 : 32	1 : 64	1 : 128	1 : 256	1 : 512	1 : 1024
IgM	+	+	+	+	+	+	+	−	−	−
IgG	+	+	+	+	+	+	+	+	+	−

**Table 4:** Results of IgM detection in clinical samples by ICS and gold standard.

Experiment	Comparative method results		Total
	Positive	Negative	
Pending evaluation results			
Positive	241	0	241
Negative	4	837	841

Total	245	837	1082

**Table 5:** Results of IgG detection in clinical samples by ICS and gold standard.

Experiment	Comparative method results		Experiment
	Positive	Negative	
Pending evaluation results			
Positive	167	12	179
Negative	4	899	903

Total	171	911	1082
